# 2-[7-(3,5-Dibromo-2-hy­droxy­phen­yl)-6-eth­oxy­carbonyl-2-oxo-5*H*-2,3,6,7-tetra­hydro­thio­pyrano[2,3-*d*][1,3]thia­zol-6-yl]acetic acid ethanol monosolvate

**DOI:** 10.1107/S1600536812035325

**Published:** 2012-08-15

**Authors:** M. Kowiel, N. Zelisko, D. Atamanyuk, R. Lesyk, A. K. Gzella

**Affiliations:** a Department of Organic Chemistry, Poznan University of Medical Sciences, ul. Grunwaldzka 6, 60-780 Poznań, Poland; b Department of Pharmaceutical, Organic and Bioorganic Chemistry, Danylo Halytsky Lviv National Medical University, Pekarska 69, Lviv, 79010, Ukraine; c Faculty of Pharmacy, Ludwik Rydygier Collegium Medicum in Bydgoszcz, Nicolaus Copernicus University in Toruń, ul. M. Curie Skłodowskiej 9, 85-094 Bydgoszcz, Poland

## Abstract

The title compound, C_17_H_15_Br_2_NO_6_S_2_·C_2_H_5_OH, is the esterification reaction product of 2-(8,10-dibromo-2,6-dioxo-3,5,5a,11*b*-tetra­hydro-2*H*,6*H*-chromeno[4′,3′:4,5]thio­pyrano[2,3-*d*]thia­zol-5a-yl)acetic acid. Cleavage of the lactone ring and formation of eth­oxy­carbonyl and hy­droxy groups from its structural elements were observed. On the other hand, the carb­oxy­methyl group was not esterified. The H atom and carb­oxy­methyl group, both at stereogenic centres, show a *cis* conformation. The six-membered dihydro­thio­pyran ring adopts a half-chair conformation. All NH and OH groups participate in the three-dimensional hydrogen-bond network, which is additionally strengthened by C—H⋯O and C—H⋯S inter­actions. Intramolecular O—H⋯Br and C—H⋯O interactions also occur.

## Related literature

For the biological activity of 4-thia­zolidinone and thio­pyrano[2,3-*d*]thia­zole-2-one derivatives, see: Lesyk & Zimenkovsky (2004 ▶); Lesyk *et al.* (2011 ▶); Kaminskyy *et al.* (2011 ▶); Matiychuk *et al.* (2008 ▶); Lesyk *et al.* (2006 ▶); Atamanyuk *et al.* (2008 ▶). For ring conformation analysis, see: Cremer & Pople (1975 ▶). For bond-length data, see: Allen *et al.* (1987 ▶).
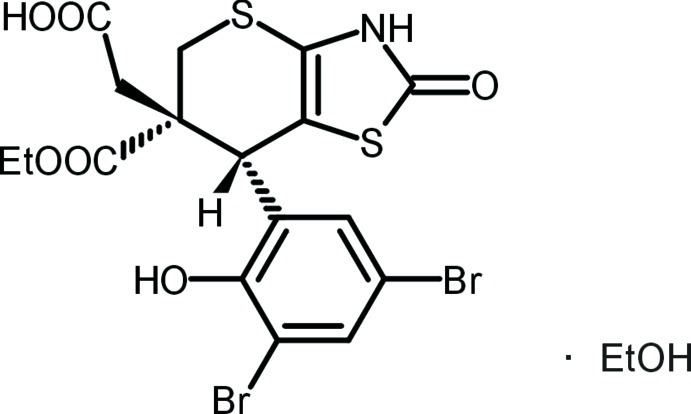


## Experimental

### 

#### Crystal data

C_17_H_15_Br_2_NO_6_S_2_·C_2_H_6_O
*M* *_r_* = 599.31
Monoclinic, 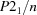

*a* = 16.8176 (9) Å
*b* = 8.1654 (4) Å
*c* = 18.3841 (9) Å
β = 113.303 (6)°
*V* = 2318.6 (2) Å^3^
*Z* = 4
Mo *K*α radiation
μ = 3.72 mm^−1^
*T* = 130 K
0.45 × 0.40 × 0.25 mm

#### Data collection

Oxford Diffraction Xcalibur Atlas diffractometer
Absorption correction: multi-scan (*CrysAlis PRO*; Oxford Diffraction, 2009 ▶) *T*_min_ = 0.761, *T*_max_ = 1.000
15312 measured reflections
5539 independent reflections
4500 reflections with *I* > 2σ(*I*)
*R*_int_ = 0.022

#### Refinement

*R*[*F* ^2^ > 2σ(*F* ^2^)] = 0.027
*wR*(*F* ^2^) = 0.073
*S* = 1.09
5539 reflections
298 parameters
H atoms treated by a mixture of independent and constrained refinement
Δρ_max_ = 1.08 e Å^−3^
Δρ_min_ = −0.87 e Å^−3^

### 

Data collection: *CrysAlis PRO* (Oxford Diffraction, 2009 ▶); cell refinement: *CrysAlis PRO*; data reduction: *CrysAlis PRO*; program(s) used to solve structure: *SHELXS97* (Sheldrick, 2008 ▶); program(s) used to refine structure: *SHELXL97* (Sheldrick, 2008 ▶); molecular graphics: *ORTEP-3 for Windows* (Farrugia, 1997 ▶); software used to prepare material for publication: *WinGX* (Farrugia, 1999 ▶) and *PLATON* (Spek, 2009 ▶).

## Supplementary Material

Crystal structure: contains datablock(s) I, global. DOI: 10.1107/S1600536812035325/bt5997sup1.cif

Structure factors: contains datablock(s) I. DOI: 10.1107/S1600536812035325/bt5997Isup2.hkl

Additional supplementary materials:  crystallographic information; 3D view; checkCIF report

## Figures and Tables

**Table 1:** Hydrogen-bond geometry (Å, °)

*D*—H⋯*A*	*D*—H	H⋯*A*	*D*⋯*A*	*D*—H⋯*A*
O26—H26⋯Br1	0.98 (3)	2.55 (3)	3.1181 (15)	117 (2)
C6—H6*A*⋯O13	0.97	2.44	3.033 (2)	119
N3—H3⋯O27^i^	0.89 (2)	1.83 (2)	2.713 (2)	171 (3)
O14—H14⋯O13^ii^	0.85 (3)	1.80 (3)	2.645 (2)	171 (3)
O26—H26⋯O16^iii^	0.98 (3)	1.96 (3)	2.7800 (19)	139 (2)
O27—H27⋯O10	0.87 (3)	1.86 (3)	2.724 (2)	174 (2)
C6—H6*A*⋯S1^iv^	0.97	2.75	3.5659 (17)	142
C23—H23⋯O10^v^	0.93	2.36	3.253 (2)	161

Symmetry codes: (i) 
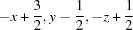
; (ii) 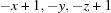; (iii) 
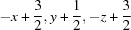
; (iv) 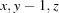; (v) 
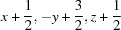
.

**Fig. 1.**
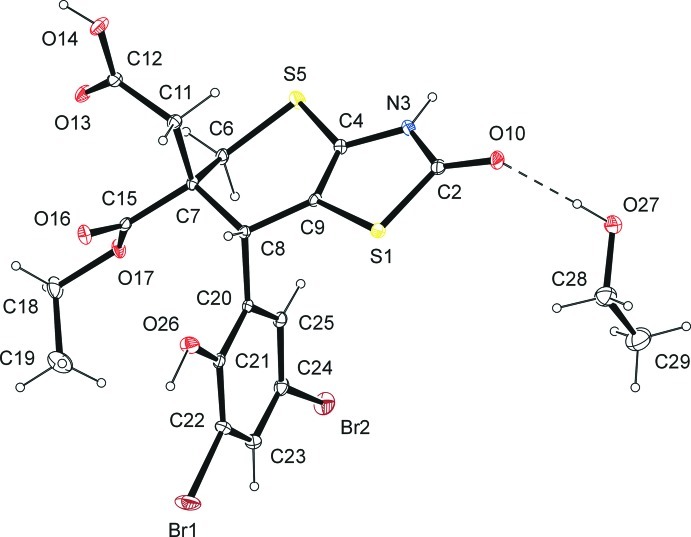
The molecular structure of (I) showing the atomic labelling scheme. Non-H atoms are drawn as 30% probability displacement ellipsoids; H atoms are shown as small spheres of arbitrary radius.

**Fig. 2.**
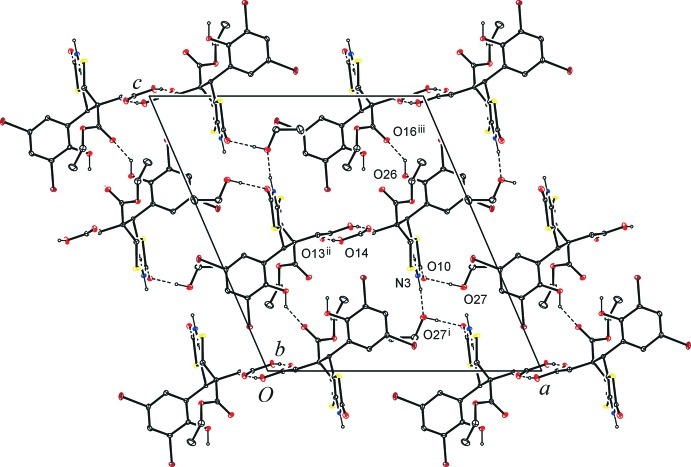
The hydrogen bonding in the title crystal structure. Symmetry codes: (i) 1.5–x, -1/2+y, 0.5–z; (ii) 1–x, –y, 1–z; (iii) 1.5–x, 1/2+y, 1.5–z. H atoms not involved in hydrogen bonds have been ommitted for clarity.

## supplementary crystallographic information

### Comment

The prominent success in thiazolidinone field is related to 4-thiazolidinone derivatives. Anticonvulsant, sedative, antidepressant, anti-inflammatory, antihypertensive, antihistaminic and anticancer activities are a few among many other biological responses shown by this scaffold (Lesyk & Zimenkovsky, 2004; Lesyk *et al.*, 2011). Among mentioned heterocycles fused heterocyclic systems, particularly thiopyrano[2,3-*d*]thiazole-2-ones possess a special interest as cyclic isosteric mimics of their synthetic precursors namely 4-thiazolidones without Michael accepting functionalities (Kaminskyy *et al.*, 2011; Matiychuk *et al.*, 2008). Fixation of highly active 5-arylidene-4-thiazolidinone in thiopyranothiazole system usually allows save the activity vector and opens up new possibilities of obtained derivatives optimization. Following the fact of anticancer activity discovery for various thiopyrano[2,3-*d*]thiazole-2-ones (Lesyk *et al.*, 2006; Atamanyuk *et al.*, 2008) the introduction of exocyclic carboxylic group into the mentioned heterocycles can be considered as on the way of lead-structures optimization. This prompted us to synthesize title compound, (I).

The molecular structure of compound (I) and the atom-labelling scheme is illustrated in Fig. 1.

The X-ray analysis showed that the crystal exists as ethanolic solvate. The asymmetric part of the unit cell contains one molecule of the compound (I) (solute) and one molecule of ethanol (solvent).

The studies on the structure of (I) showed that refluxing of 2-(8,10-dibromo-2,6-dioxo-3,5,5a,11*b*-tetrahydro-2*H*,6*H*-chromeno[4',3':4,5]thiopyrano[2,3-*d*]thiazol-5a-yl)acetic acid for three hours in ethanol resulted in the cleavage of the lactone ring and formation of an ethoxycarbonyl moiety from its structural elements. On the other hand, the carboxymethyl group was not esterified under these conditions.

Investigations of the geometry of dihydrothiopyrano[2,3-*d*]thiazol-2-one showed that the six-membered dihydrothiopyran ring has a half-chair conformation [Cremer & Pople (1975) puckering parameters: *Q* = 0.5136 (19) Å, *Θ* = 49.7 (2)°, *φ* = 268.5 (2)°].

The C4═C9 bond length of 1.342 (2) Å confirmed the presence of a double bond between these carbons.

The C2—N3 interatomic distance of 1.356 (3) Å in the thiazol-2-one moiety is lengthened of about 7σ relative to the normal (*O═* )C—NH bond length [1.331 (2) Å] of secondary amide group of *γ*-lactam (Allen *et al.*, 1987).

The carboxymethyl and ethoxycarbonyl groups at C7 atom of dihydrothiopyran ring are in an axial and equatorial positions, respectively, while the 3,4-dibromo-2-hydroxyphenyl substituent at C8 atom is in a pseudoaxial position.

The carboxymethyl and ethoxycarbonyl groups are *trans* and *cis*, respectively, relative to the 3,4-dibromo-2-hydroxyphenyl substituent. The torsion angles C11—C7—C8—C20 and C15—C7—C8—C20 are 159.65 (15) and 45.32 (19)°, respectively.

The planar carboxymethyl and phenyl groups are approximately perpendicular to the least squares plane of the dihydrothiopyran ring; the dihedral angles are 83.10 (6) and 86.47 (6)°, respectively. The C12═O13 carbonyl group of the carboxymethyl substituent is synperiplanar (+*sp*) relative to the C7—C11 bond [torsion angle C7—C11—C12—O13: 2.3 (2)°]. On the other hand, the C11—C12 bond is antiperiplanar (-*ap*) in relation to the C7—C8 bond [torsion angle: C8—C7—C11—C12: -168.91 (14)°]. The C21 atom of the 3,4-dibromo-2-hydroxyphenyl substituent, at which the hydroxy group is attached, is anticlinal relative to the C7—C8 bond (-*ac*) [torsion angle C7—C8—C20—C21: -108.48 (18)°].

The flat fragment of the ethoxycarbonyl group, consisted of C15,O16,O17, and C18 atoms, forms a 55.18 (6)° dihedral angle with the best plane of dihydrothiopyran ring. The remaining C19 atom of the ethoxycarbonyl group, projected away of 1.367 (4) Å from the above atoms plane, is synclinal relative to the C15—O17 bond [torsion angle C15—O17—C18—C19: -81.1 (2)°]. Moreover, the C15═O16 carbonyl group is synclinal in relation to the C7—C8 bond [torsion angle C8—C7—C15—O16: 76.5 (2)°]. However, the torsion angles C15—O17—C18—C19 and C8—C7—C15—O16 are of different signs.

The molecules of (I) are interconnected with a screw axis and are linked by hydrogen bonds O26—H26···O16^iii^ in chains (Table 1, Fig. 2). The neighbouring chains exist in antiparallel arrangement and are connected by hydrogen bonds O14—H14···.O13^ii^ in layers growing parallel to the (-101) plane (Table 1, Fig. 2). The ethanol molecules form hydrogen bonding O27—H27···O10 and N3—H3···O27^i^ (Table 1, Fig. 2) being both proton donors and acceptors. They link the molecules from neighbouring layers that results in formation of a three-dimensional lattice of hydrogen bonds in the crystal.

### Experimental

An equimolar mixture of 5-(2-hydroxy-3,5-dibromobenzylidene)-4-thioxo-2-thiazolidinone, itaconic anhydride and pinch of hydroquinone (2–3 mg, for preventing of polymerization) in acetic acid was refluxed for 2 hrs. The product formed was filtered, washed, dried and re-crystallized from mixture DMF–AcOH. Obtained 2-(8,10-dibromo-2,6-dioxo-3,5,5a,11*b*-tetrahydro-2*H*,6*H*-chromeno[4',3':4,5]thiopyrano[2,3-*d*]thiazol-5a-yl)acetic acid was refluxed in ethanol for 3 hrs. The product formed was filtered, washed, dried and recrystallized from ethanol.

### Refinement

Except for the amide and hydroxy H atoms which were refined freely the remaining H atoms were positioned into the idealized positions and were refined within the riding model approximation: C_methyl_—H = 0.96 Å, C_methylene_—H = 0.97 Å, C_methine_—H = 0.98 Å, C(*sp*^2^)—H = 0.93 Å; *U*_iso_(H) = 1.2*U*_eq_(C) or 1.5*U*_eq_(C) for methyl H. The methyl groups were refined as rigid groups which were allowed to rotate. The largest peaks and holes in the *ΔF* Fourier map are within 1.0 Å of the Br1 and Br2 atom sites.

### Crystal data

**Table:**

C_17_H_15_Br_2_NO_6_S_2_·C_2_H_6_O	*F*(000) = 1200
*M**_r_* = 599.31	*D*_x_ = 1.717 Mg m^−^^3^
Monoclinic, *P*2_1_/*n*	Melting point = 510–512 K
Hall symbol: -P 2yn	Mo *K*α radiation, λ = 0.71073 Å
*a* = 16.8176 (9) Å	Cell parameters from 6534 reflections
*b* = 8.1654 (4) Å	θ = 2.4–29.1°
*c* = 18.3841 (9) Å	µ = 3.72 mm^−^^1^
β = 113.303 (6)°	*T* = 130 K
*V* = 2318.6 (2) Å^3^	Block, colourless
*Z* = 4	0.45 × 0.40 × 0.25 mm

### Data collection

**Table:**

Oxford Diffraction Xcalibur Atlas diffractometer	5539 independent reflections
Radiation source: Enhance (Mo) X-ray Source	4500 reflections with *I* > 2σ(*I*)
Graphite monochromator	*R*_int_ = 0.022
ω scans	θ_max_ = 29.1°, θ_min_ = 2.4°
Absorption correction: multi-scan (*CrysAlis PRO*; Oxford Diffraction, 2009)	*h* = −22→22
*T*_min_ = 0.761, *T*_max_ = 1.000	*k* = −10→11
15312 measured reflections	*l* = −24→24

### Refinement

**Table:**

Refinement on *F*^2^	Primary atom site location: structure-invariant direct methods
Least-squares matrix: full	Secondary atom site location: difference Fourier map
*R*[*F*^2^ > 2σ(*F*^2^)] = 0.027	Hydrogen site location: inferred from neighbouring sites
*wR*(*F*^2^) = 0.073	H atoms treated by a mixture of independent and constrained refinement
*S* = 1.09	*w* = 1/[σ^2^(*F*_o_^2^) + (0.044*P*)^2^] where *P* = (*F*_o_^2^ + 2*F*_c_^2^)/3
5539 reflections	(Δ/σ)_max_ = 0.002
298 parameters	Δρ_max_ = 1.08 e Å^−^^3^
0 restraints	Δρ_min_ = −0.87 e Å^−^^3^

### Special details

**Table:**

Geometry. All e.s.d.'s (except the e.s.d. in the dihedral angle between two l.s. planes) are estimated using the full covariance matrix. The cell e.s.d.'s are taken into account individually in the estimation of e.s.d.'s in distances, angles and torsion angles; correlations between e.s.d.'s in cell parameters are only used when they are defined by crystal symmetry. An approximate (isotropic) treatment of cell e.s.d.'s is used for estimating e.s.d.'s involving l.s. planes.
Refinement. Refinement of *F*^2^ against ALL reflections. The weighted *R*-factor *wR* and goodness of fit *S* are based on *F*^2^, conventional *R*-factors *R* are based on *F*, with *F* set to zero for negative *F*^2^. The threshold expression of *F*^2^ > σ(*F*^2^) is used only for calculating *R*-factors(gt) *etc*. and is not relevant to the choice of reflections for refinement. *R*-factors based on *F*^2^ are statistically about twice as large as those based on *F*, and *R*- factors based on ALL data will be even larger.

### Fractional atomic coordinates and isotropic or equivalent isotropic displacement parameters (Å^2^)

**Table:**

	*x*	*y*	*z*	*U*_iso_*/*U*_eq_	
Br1	0.996319 (13)	0.62878 (3)	0.839566 (12)	0.03491 (8)	
Br2	1.074501 (12)	0.35750 (3)	0.592151 (13)	0.02712 (7)	
S1	0.75077 (3)	0.80961 (6)	0.48078 (3)	0.01618 (11)	
C2	0.71841 (11)	0.8163 (2)	0.37660 (11)	0.0157 (4)	
N3	0.69780 (10)	0.66259 (19)	0.34724 (9)	0.0148 (3)	
H3	0.6864 (14)	0.639 (3)	0.2971 (14)	0.017 (6)*	
C4	0.70683 (11)	0.5421 (2)	0.40400 (10)	0.0126 (4)	
S5	0.68259 (3)	0.33898 (6)	0.37237 (3)	0.01932 (11)	
C6	0.72417 (12)	0.2434 (2)	0.46983 (11)	0.0144 (4)	
H6A	0.7010	0.1332	0.4647	0.017*	
H6B	0.7866	0.2345	0.4884	0.017*	
C7	0.70239 (11)	0.3352 (2)	0.53289 (10)	0.0115 (3)	
C8	0.74732 (10)	0.5062 (2)	0.55304 (10)	0.0104 (3)	
H8	0.7183	0.5682	0.5812	0.013*	
C9	0.73311 (11)	0.5980 (2)	0.47848 (10)	0.0115 (4)	
O10	0.71500 (8)	0.94164 (17)	0.33849 (8)	0.0213 (3)	
C11	0.60359 (11)	0.3610 (2)	0.50519 (11)	0.0140 (4)	
H11A	0.5927	0.4331	0.5422	0.017*	
H11B	0.5816	0.4140	0.4538	0.017*	
C12	0.55580 (11)	0.2028 (2)	0.49939 (11)	0.0156 (4)	
O13	0.59224 (8)	0.07109 (16)	0.51734 (8)	0.0178 (3)	
O14	0.47090 (8)	0.22172 (18)	0.47434 (9)	0.0224 (3)	
H14	0.4454 (16)	0.132 (3)	0.4748 (16)	0.037 (8)*	
C15	0.73196 (11)	0.2392 (2)	0.61104 (11)	0.0132 (4)	
O16	0.70277 (8)	0.26568 (16)	0.66029 (7)	0.0182 (3)	
O17	0.79311 (8)	0.12991 (15)	0.61807 (8)	0.0171 (3)	
C18	0.82447 (13)	0.0290 (3)	0.69041 (12)	0.0246 (5)	
H18A	0.8501	−0.0706	0.6807	0.030*	
H18B	0.7759	−0.0016	0.7033	0.030*	
C19	0.89052 (17)	0.1187 (3)	0.75959 (15)	0.0419 (7)	
H19A	0.8633	0.2095	0.7738	0.063*	
H19B	0.9361	0.1583	0.7452	0.063*	
H19C	0.9143	0.0455	0.8038	0.063*	
C20	0.84402 (11)	0.4998 (2)	0.60762 (10)	0.0112 (3)	
C21	0.87065 (11)	0.5535 (2)	0.68594 (10)	0.0133 (4)	
C22	0.95879 (12)	0.5481 (3)	0.73445 (10)	0.0186 (4)	
C23	1.02008 (11)	0.4888 (3)	0.70848 (11)	0.0202 (4)	
H23	1.0783	0.4837	0.7423	0.024*	
C24	0.99208 (12)	0.4375 (2)	0.63082 (12)	0.0173 (4)	
C25	0.90546 (11)	0.4444 (2)	0.58003 (11)	0.0143 (4)	
H25	0.8883	0.4122	0.5275	0.017*	
O26	0.80868 (9)	0.61034 (17)	0.70964 (8)	0.0207 (3)	
H26	0.8325 (17)	0.644 (3)	0.7654 (17)	0.046 (8)*	
O27	0.84716 (10)	1.0647 (2)	0.30489 (9)	0.0325 (4)	
H27	0.8036 (16)	1.032 (3)	0.3155 (15)	0.041 (7)*	
C28	0.91066 (14)	1.1225 (3)	0.37811 (13)	0.0307 (5)	
H28A	0.9057	1.0634	0.4219	0.037*	
H28B	0.9016	1.2380	0.3845	0.037*	
C29	0.99888 (15)	1.0965 (3)	0.37809 (17)	0.0432 (7)	
H29A	1.0085	0.9815	0.3744	0.065*	
H29B	1.0419	1.1392	0.4262	0.065*	
H29C	1.0027	1.1523	0.3336	0.065*	

### Atomic displacement parameters (Å^2^)

**Table:**

	*U*^11^	*U*^22^	*U*^33^	*U*^12^	*U*^13^	*U*^23^
Br1	0.01994 (11)	0.0685 (2)	0.01402 (10)	−0.00743 (10)	0.00428 (8)	−0.01027 (10)
Br2	0.01891 (11)	0.03218 (14)	0.03580 (13)	0.00589 (9)	0.01672 (9)	0.00288 (10)
S1	0.0194 (2)	0.0091 (2)	0.0160 (2)	−0.00223 (18)	0.00270 (18)	0.00152 (18)
C2	0.0100 (8)	0.0176 (10)	0.0185 (9)	0.0012 (7)	0.0047 (7)	0.0051 (8)
N3	0.0163 (8)	0.0157 (8)	0.0126 (7)	0.0013 (6)	0.0059 (6)	0.0032 (6)
C4	0.0135 (8)	0.0123 (9)	0.0132 (8)	0.0003 (7)	0.0064 (7)	0.0008 (7)
S5	0.0323 (3)	0.0126 (2)	0.0124 (2)	−0.0027 (2)	0.00818 (19)	−0.00275 (18)
C6	0.0185 (9)	0.0100 (9)	0.0158 (8)	−0.0028 (7)	0.0079 (7)	−0.0002 (7)
C7	0.0128 (8)	0.0092 (8)	0.0132 (8)	−0.0004 (7)	0.0057 (7)	0.0013 (7)
C8	0.0116 (8)	0.0093 (9)	0.0109 (8)	−0.0008 (7)	0.0051 (6)	−0.0008 (7)
C9	0.0108 (8)	0.0076 (8)	0.0153 (8)	−0.0006 (7)	0.0045 (7)	0.0007 (7)
O10	0.0198 (7)	0.0173 (7)	0.0241 (7)	−0.0006 (6)	0.0058 (6)	0.0102 (6)
C11	0.0137 (8)	0.0120 (9)	0.0165 (9)	−0.0002 (7)	0.0062 (7)	0.0002 (7)
C12	0.0138 (8)	0.0191 (10)	0.0140 (8)	−0.0025 (8)	0.0057 (7)	−0.0006 (8)
O13	0.0160 (6)	0.0110 (7)	0.0258 (7)	−0.0024 (5)	0.0076 (5)	0.0003 (6)
O14	0.0144 (7)	0.0158 (7)	0.0340 (8)	−0.0040 (6)	0.0064 (6)	0.0028 (6)
C15	0.0130 (8)	0.0094 (9)	0.0164 (8)	−0.0032 (7)	0.0050 (7)	0.0008 (7)
O16	0.0245 (7)	0.0170 (7)	0.0161 (6)	0.0012 (6)	0.0114 (6)	0.0035 (5)
O17	0.0189 (6)	0.0149 (7)	0.0186 (7)	0.0055 (5)	0.0087 (5)	0.0077 (5)
C18	0.0277 (11)	0.0191 (11)	0.0273 (11)	0.0066 (9)	0.0112 (9)	0.0122 (9)
C19	0.0368 (13)	0.0460 (16)	0.0305 (13)	0.0038 (11)	0.0001 (11)	0.0122 (11)
C20	0.0132 (8)	0.0084 (8)	0.0120 (8)	−0.0019 (7)	0.0051 (7)	0.0016 (7)
C21	0.0147 (8)	0.0132 (9)	0.0139 (8)	−0.0030 (7)	0.0075 (7)	0.0007 (7)
C22	0.0176 (9)	0.0250 (11)	0.0104 (8)	−0.0043 (8)	0.0024 (7)	−0.0002 (8)
C23	0.0123 (9)	0.0271 (11)	0.0183 (9)	−0.0019 (8)	0.0028 (7)	0.0053 (8)
C24	0.0141 (8)	0.0182 (10)	0.0227 (9)	0.0013 (8)	0.0106 (7)	0.0036 (8)
C25	0.0158 (9)	0.0120 (9)	0.0160 (8)	−0.0018 (7)	0.0072 (7)	0.0000 (7)
O26	0.0204 (7)	0.0289 (8)	0.0149 (7)	−0.0018 (6)	0.0091 (6)	−0.0064 (6)
O27	0.0262 (8)	0.0515 (11)	0.0188 (7)	−0.0123 (8)	0.0079 (6)	0.0049 (7)
C28	0.0312 (12)	0.0357 (14)	0.0252 (11)	−0.0081 (10)	0.0111 (9)	−0.0066 (10)
C29	0.0270 (12)	0.0459 (16)	0.0511 (16)	−0.0037 (11)	0.0094 (11)	0.0067 (13)

### Geometric parameters (Å, º)

**Table:**

Br1—C22	1.8980 (18)	C15—O17	1.329 (2)
Br2—C24	1.9061 (19)	O17—C18	1.473 (2)
S1—C9	1.7510 (18)	C18—C19	1.507 (3)
S1—C2	1.773 (2)	C18—H18A	0.9700
C2—O10	1.229 (2)	C18—H18B	0.9700
C2—N3	1.356 (3)	C19—H19A	0.9600
N3—C4	1.398 (2)	C19—H19B	0.9600
N3—H3	0.88 (2)	C19—H19C	0.9600
C4—C9	1.342 (2)	C20—C25	1.394 (3)
C4—S5	1.7515 (19)	C20—C21	1.398 (2)
S5—C6	1.8212 (19)	C21—O26	1.361 (2)
C6—C7	1.541 (3)	C21—C22	1.396 (2)
C6—H6A	0.9700	C22—C23	1.383 (3)
C6—H6B	0.9700	C23—C24	1.380 (3)
C7—C15	1.536 (2)	C23—H23	0.9300
C7—C11	1.548 (2)	C24—C25	1.386 (2)
C7—C8	1.561 (2)	C25—H25	0.9300
C8—C9	1.496 (2)	O26—H26	0.98 (3)
C8—C20	1.537 (2)	O27—C28	1.427 (3)
C8—H8	0.9800	O27—H27	0.87 (3)
C11—C12	1.503 (3)	C28—C29	1.499 (3)
C11—H11A	0.9700	C28—H28A	0.9700
C11—H11B	0.9700	C28—H28B	0.9700
C12—O13	1.217 (2)	C29—H29A	0.9600
C12—O14	1.325 (2)	C29—H29B	0.9600
O14—H14	0.85 (3)	C29—H29C	0.9600
C15—O16	1.208 (2)		
			
C9—S1—C2	91.59 (9)	C15—O17—C18	116.76 (15)
O10—C2—N3	126.65 (18)	O17—C18—C19	111.79 (18)
O10—C2—S1	124.54 (16)	O17—C18—H18A	109.3
N3—C2—S1	108.81 (14)	C19—C18—H18A	109.3
C2—N3—C4	114.77 (16)	O17—C18—H18B	109.3
C2—N3—H3	122.1 (14)	C19—C18—H18B	109.3
C4—N3—H3	122.6 (14)	H18A—C18—H18B	107.9
C9—C4—N3	114.66 (17)	C18—C19—H19A	109.5
C9—C4—S5	126.90 (15)	C18—C19—H19B	109.5
N3—C4—S5	118.44 (13)	H19A—C19—H19B	109.5
C4—S5—C6	97.51 (8)	C18—C19—H19C	109.5
C7—C6—S5	114.75 (12)	H19A—C19—H19C	109.5
C7—C6—H6A	108.6	H19B—C19—H19C	109.5
S5—C6—H6A	108.6	C25—C20—C21	119.64 (15)
C7—C6—H6B	108.6	C25—C20—C8	121.24 (15)
S5—C6—H6B	108.6	C21—C20—C8	119.12 (15)
H6A—C6—H6B	107.6	O26—C21—C22	124.10 (16)
C15—C7—C6	111.71 (14)	O26—C21—C20	117.57 (15)
C15—C7—C11	106.57 (14)	C22—C21—C20	118.33 (16)
C6—C7—C11	111.24 (14)	C23—C22—C21	122.58 (17)
C15—C7—C8	106.69 (13)	C23—C22—Br1	118.67 (13)
C6—C7—C8	112.13 (14)	C21—C22—Br1	118.74 (14)
C11—C7—C8	108.21 (14)	C24—C23—C22	117.86 (16)
C9—C8—C20	111.07 (14)	C24—C23—H23	121.1
C9—C8—C7	110.11 (14)	C22—C23—H23	121.1
C20—C8—C7	114.34 (13)	C23—C24—C25	121.48 (18)
C9—C8—H8	107.0	C23—C24—Br2	119.28 (14)
C20—C8—H8	107.0	C25—C24—Br2	119.23 (15)
C7—C8—H8	107.0	C24—C25—C20	120.06 (17)
C4—C9—C8	129.31 (16)	C24—C25—H25	120.0
C4—C9—S1	110.15 (14)	C20—C25—H25	120.0
C8—C9—S1	120.54 (13)	C21—O26—H26	112.4 (16)
C12—C11—C7	112.39 (15)	C28—O27—H27	105.8 (17)
C12—C11—H11A	109.1	O27—C28—C29	108.9 (2)
C7—C11—H11A	109.1	O27—C28—H28A	109.9
C12—C11—H11B	109.1	C29—C28—H28A	109.9
C7—C11—H11B	109.1	O27—C28—H28B	109.9
H11A—C11—H11B	107.9	C29—C28—H28B	109.9
O13—C12—O14	123.74 (17)	H28A—C28—H28B	108.3
O13—C12—C11	122.87 (16)	C28—C29—H29A	109.5
O14—C12—C11	113.39 (16)	C28—C29—H29B	109.5
C12—O14—H14	112.1 (17)	H29A—C29—H29B	109.5
O16—C15—O17	125.14 (17)	C28—C29—H29C	109.5
O16—C15—C7	122.17 (16)	H29A—C29—H29C	109.5
O17—C15—C7	112.67 (15)	H29B—C29—H29C	109.5
			
C9—S1—C2—O10	178.68 (17)	C7—C11—C12—O13	2.3 (3)
C9—S1—C2—N3	−0.71 (14)	C7—C11—C12—O14	−178.81 (15)
O10—C2—N3—C4	−179.39 (17)	C6—C7—C15—O16	−160.59 (16)
S1—C2—N3—C4	−0.01 (19)	C11—C7—C15—O16	−38.9 (2)
C2—N3—C4—C9	1.0 (2)	C8—C7—C15—O16	76.5 (2)
C2—N3—C4—S5	−179.55 (13)	C6—C7—C15—O17	20.9 (2)
C9—C4—S5—C6	−10.25 (18)	C11—C7—C15—O17	142.56 (15)
N3—C4—S5—C6	170.43 (14)	C8—C7—C15—O17	−102.00 (16)
C4—S5—C6—C7	41.76 (14)	O16—C15—O17—C18	2.8 (3)
S5—C6—C7—C15	174.57 (12)	C7—C15—O17—C18	−178.74 (15)
S5—C6—C7—C11	55.62 (17)	C15—O17—C18—C19	−81.1 (2)
S5—C6—C7—C8	−65.71 (16)	C9—C8—C20—C25	−52.6 (2)
C15—C7—C8—C9	171.17 (14)	C7—C8—C20—C25	72.8 (2)
C6—C7—C8—C9	48.57 (19)	C9—C8—C20—C21	126.18 (18)
C11—C7—C8—C9	−74.49 (18)	C7—C8—C20—C21	−108.48 (18)
C15—C7—C8—C20	45.32 (19)	C25—C20—C21—O26	178.33 (16)
C6—C7—C8—C20	−77.29 (18)	C8—C20—C21—O26	−0.5 (2)
C11—C7—C8—C20	159.65 (15)	C25—C20—C21—C22	−0.7 (3)
N3—C4—C9—C8	178.26 (16)	C8—C20—C21—C22	−179.45 (16)
S5—C4—C9—C8	−1.1 (3)	O26—C21—C22—C23	179.74 (18)
N3—C4—C9—S1	−1.5 (2)	C20—C21—C22—C23	−1.3 (3)
S5—C4—C9—S1	179.11 (11)	O26—C21—C22—Br1	−1.4 (3)
C20—C8—C9—C4	111.4 (2)	C20—C21—C22—Br1	177.50 (14)
C7—C8—C9—C4	−16.3 (3)	C21—C22—C23—C24	1.7 (3)
C20—C8—C9—S1	−68.83 (18)	Br1—C22—C23—C24	−177.11 (15)
C7—C8—C9—S1	163.48 (12)	C22—C23—C24—C25	−0.1 (3)
C2—S1—C9—C4	1.28 (14)	C22—C23—C24—Br2	179.24 (15)
C2—S1—C9—C8	−178.54 (14)	C23—C24—C25—C20	−1.8 (3)
C15—C7—C11—C12	−54.49 (19)	Br2—C24—C25—C20	178.81 (14)
C6—C7—C11—C12	67.49 (19)	C21—C20—C25—C24	2.2 (3)
C8—C7—C11—C12	−168.91 (14)	C8—C20—C25—C24	−179.02 (16)

### Hydrogen-bond geometry (Å, º)

**Table:**

*D*—H···*A*	*D*—H	H···*A*	*D*···*A*	*D*—H···*A*
O26—H26···Br1	0.98 (3)	2.55 (3)	3.1181 (15)	117 (2)
C6—H6*A*···O13	0.97	2.44	3.033 (2)	119
N3—H3···O27^i^	0.89 (2)	1.83 (2)	2.713 (2)	171 (3)
O14—H14···O13^ii^	0.85 (3)	1.80 (3)	2.645 (2)	171 (3)
O26—H26···O16^iii^	0.98 (3)	1.96 (3)	2.7800 (19)	139 (2)
O27—H27···O10	0.87 (3)	1.86 (3)	2.724 (2)	174 (2)
C6—H6*A*···S1^iv^	0.97	2.75	3.5659 (17)	142
C23—H23···O10^v^	0.93	2.36	3.253 (2)	161

Symmetry codes: (i) −*x*+3/2, *y*−1/2, −*z*+1/2; (ii) −*x*+1, −*y*, −*z*+1; (iii) −*x*+3/2, *y*+1/2, −*z*+3/2; (iv) *x*, *y*−1, *z*; (v) *x*+1/2, −*y*+3/2, *z*+1/2.
